# Disrupted Vitamin D Metabolism in Hepatocellular Carcinoma: Free and Bioavailable 25(OH)D as Novel Biomarkers of Hepatic Reserve and Clinical Risk

**DOI:** 10.3390/molecules31020273

**Published:** 2026-01-13

**Authors:** Joško Osredkar, Matej Rakusa, Aleš Jerin, Borut Štabuc, Martin Zaplotnik, Saša Štupar, Darko Siuka

**Affiliations:** 1 Institute of Clinical Chemistry and Biochemistry, University Medical Centre Ljubljana, Zaloška c 2, 1000 Ljubljana, Slovenia; josko.osredkar@kclj.si (J.O.); ales.jerin@kclj.si (A.J.); 2 Faculty of Pharmacy, University of Ljubljana, Aškerčeva 7, 1000 Ljubljana, Slovenia; 3 Faculty of Medicine, University of Ljubljana, Vrazov trg 2, 1000 Ljubljana, Sloveniaborut.stabuc@kclj.si (B.Š.); martin.zaplotnik@kclj.si (M.Z.); sasa.stupar@kclj.si (S.Š.); 4 Department of Endocrinology, Diabetes and Metabolic Disease, University Medical Centre Ljubljana, Zaloška c 2, 1000 Ljubljana, Slovenia; 5 Department of Gastroenterology, University Medical Centre Ljubljana, Zaloška c 2, 1000 Ljubljana, Slovenia

**Keywords:** hepatocellular carcinoma, vitamin D metabolism, 25-hydroxyvitamin D, free vitamin D, bioavailable vitamin D, vitamin D-binding protein, albumin, Child-Pugh score, BCLC staging, biomarkers

## Abstract

Background: Although total 25-hydroxyvitamin D (25(OH)D) measurements may not accurately reflect functional vitamin D status, vitamin D deficiency is common in hepatocellular carcinoma (HCC). The contribution of altered vitamin D-binding protein (VDBP) and albumin to impaired bioavailability is poorly characterized in liver cancer. Methods: We measured total, free, and bioavailable 25(OH)D, VDBP, and albumin in 46 HCC patients and 87 healthy controls during winter and summer. Correlations with Child–Pugh score, Barcelona Clinic Liver Cancer (BCLC) stage, and disease aetiology were evaluated. Results: HCC patients exhibited significantly lower VDBP (177.3 ± 237.0 vs. 239.9 ± 141.9 mg/L, *p* < 0.001) and albumin (35.9 ± 5.4 vs. 48.0 ± 3.9 g/L, *p* < 0.001) compared to winter controls. Total 25(OH)D was lower in HCC (39.3 ± 22.1 nmol/L) versus summer controls (75.0 ± 22.8 nmol/L, *p* < 0.001) but comparable to winter controls (*p* = 0.061). HCC patients lacked seasonal variation in vitamin D fractions, unlike the controls. VDBP negatively correlated with free (ρ = −0.606, *p* < 0.001) and bioavailable 25(OH)D (ρ = −0.541, *p* < 0.001). Child–Pugh score correlated positively with BCLC stage (ρ = 0.378, *p* = 0.012) and inversely with albumin (ρ = −0.565, *p* < 0.001). Conclusions: Free and bioavailable vitamin D are profoundly compromised in HCC, reflecting impaired hepatic synthetic function and systemic inflammation. These fractions may serve as novel metabolic biomarkers superior to total 25(OH)D for assessing vitamin D deficiency and guiding individualized supplementation strategies in patients with liver cancer.

## 1. Introduction

Vitamin D is a pleiotropic hormone with established roles in mineral homeostasis and increasingly recognized functions in immune modulation, cellular proliferation control, and cancer biology [1,2,3]. Vitamin D has well-established roles in cancer prevention and treatment, exerting antiproliferative, pro-apoptotic, anti-angiogenic and immunomodulatory effects across multiple tumour types. Recent reviews have summarized how lower circulating vitamin D levels and vitamin D receptor (VDR) pathway dysregulation are associated with increased incidence and poorer outcomes of several common cancers, and how vitamin D supplementation or vitamin D analogues may potentiate standard oncologic therapies. In hepatocellular carcinoma specifically, preclinical and translational data indicate that vitamin D signalling modulates hepatic stellate cell activation, inflammatory pathways, and tumour cell proliferation and invasiveness, supporting a potential role in both hepatocellular carcinoma (HCC) prevention and prognosis [4,5,6]. HCC, the most common primary liver malignancy, predominantly arises in cirrhotic livers subject to chronic inflammation and hepatic dysfunction—conditions that profoundly perturb vitamin D metabolism and transport [7,8,9].

At the enzymatic level, hepatic 25-hydroxylation of vitamin D is primarily mediated by microsomal CYP2R1 and mitochondrial CYP27A1, whereas subsequent 1α-hydroxylation to 1,25(OH)22D is catalysed mainly by renal and extra-renal CYP27B1, and catabolism by CYP24A1. In advanced liver disease and HCC, reduced hepatic mass, inflammation-induced downregulation of CYP2R1 and CYP27A1, and altered expression of CYP27B1 and CYP24A1 have all been implicated in impaired vitamin D activation and accelerated degradation. Future studies in HCC should therefore directly assess hepatic and, where feasible, tumour-specific expression and activity of CYP2R1, CYP27A1, CYP27B1 and CYP24A1, alongside VDR, using enzyme activity assays, quantitative PCR and immunohistochemistry to delineate the relative contributions of synthetic failure versus enhanced catabolism [10,11]. The liver is essential for two critical steps in vitamin D homeostasis: first, the 25-hydroxylation of vitamin D to generate the major circulating metabolite, 25-hydroxyvitamin D (25(OH)D); second, the synthesis of vitamin D-binding protein (VDBP) and albumin, which carry vitamin D in serum [3,12,13]. In HCC and advanced cirrhosis, both processes are compromised by reduced hepatic synthetic capacity and systemic inflammatory cytokines such as IL-6 and TNF-α, which suppress VDBP production [14,15,16].

Despite evidence suggesting that low total 25(OH)D portends a worse HCC prognosis, few studies have systematically profiled the spectrum of vitamin D metabolites—including free and bioavailable fractions—in relation to clinical stage, hepatic function, and inflammation in HCC [17,18]. This gap has direct therapeutic implications, as vitamin D supplementation regimens tailored to total 25(OH)D may be insufficient or potentially unsafe in the context of significant protein-binding alterations [19].

In circulation, approximately 85–90% of 25OHD is tightly bound to VDBP, 10–15% is loosely bound to albumin, and less than 1% circulates as truly free hormone [3,12,13,20]. Total 25OHD reflects the sum of all these fractions, whereas free 25OHD denotes the unbound fraction, and bioavailable 25OHD comprises free plus albumin-bound 25OHD, which can readily dissociate and enter cells according to the free hormone hypothesis [21,22]. In conditions characterized by hypoalbuminaemia and reduced VDBP synthesis, such as advanced liver disease and HCC, total 25OHD can therefore substantially misrepresent the biologically active vitamin D pool. Measuring or calculating free and bioavailable 25OHD alongside VDBP and albumin may thus provide a more accurate assessment of functional vitamin D status in HCC [6].

**Figure 1:**
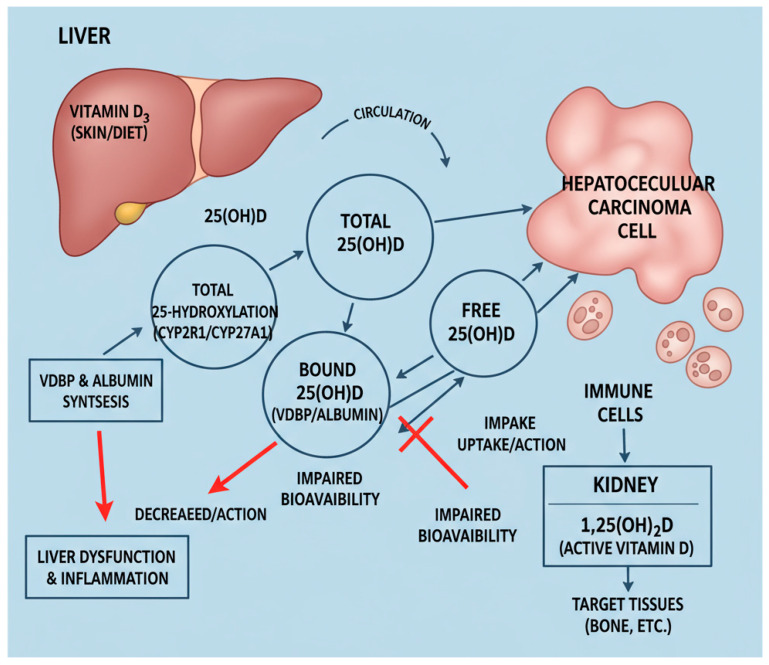
Schematic representation of altered vitamin D metabolism and transport in hepatocellular carcinoma (HCC). Under physiological conditions, vitamin D3 produced in the skin or obtained from diet is transported to the liver, where it is converted by 25-hydroxylases (mainly CYP2R1 and CYP27A1) into 25-hydroxyvitamin D (25OHD). Hepatocytes also synthesize vitamin D-binding protein (VDBP) and albumin, which bind most circulating 25OHD and thereby determine the proportions of total, free and bioavailable 25OHD. The 25OHD–VDBP/albumin complexes are subsequently filtered by the kidney, where proximal tubular CYP27B1 converts 25OHD into the active hormone 1,25(OH)_2_D, and CYP24A1 mediates catabolism. Free and bioavailable 25OHD and 1,25(OH)_2_D can enter hepatocytes, tumour cells and immune cells to activate vitamin D receptor (VDR)–mediated signalling involved in immune modulation and anti-tumour responses. In HCC, reduced hepatic mass and synthetic failure lead to a decrease in VDBP and albumin production and impaired 25-hydroxylation, while systemic inflammation further suppresses VDBP synthesis. These changes lower circulating total, free and bioavailable 25OHD and disrupt normal VDR signalling in tumour and immune cells. Solid arrows indicate physiological metabolic and transport pathways.

Vitamin D metabolism and bioavailability depend critically on intact liver function. The liver not only converts vitamin D to its main circulating form, 25OHD, but also synthesizes VDBP and albumin, the principal carrier proteins that regulate the proportion of free and bioavailable 25OHD reaching target tissues. In HCC, hepatic dysfunction and systemic inflammation disrupt these pathways, leading to a decrease in the production of VDBP and albumin, thereby diminishing vitamin D bioavailability despite serum levels that may not fully reveal the deficiency [14,15,20]. This complex interplay is summarized schematically in Figure 1.

Conventionally, vitamin D status is assessed by measuring total serum 25(OH)D. However, the “free hormone hypothesis” posits that only the unbound or bioavailable fractions of hormones can cross cell membranes and activate receptors [23,24]. In populations with low albumin and VDBP—such as patients with HCC total 25(OH)D may substantially underestimate true vitamin D deficiency [12,25,26,27]. Recent epidemiological and translational studies support the role of vitamin D in HCC prevention and possibly prognosis, yet few investigations have comprehensively characterized all vitamin D fractions or their carrier proteins in relation to clinical liver function markers [8,17,27,28,29].

The aim of this study was to systematically profile total, free, and bioavailable 25(OH)D alongside VDBP and albumin in HCC patients compared to healthy controls across seasons, and to evaluate their correlations with measures of hepatic reserve, tumour burden (BCLC stage), and disease aetiology. We hypothesized that free and bioavailable 25(OH)D, rather than total measurements alone, would better reflect functional vitamin D deficiency and correlate with clinical disease severity in HCC.

## 2. Results

### 2.1. Patient Characteristics

The HCC cohort (n = 46) consisted predominantly of male patients (39 males and 7 females) with a mean age of 71.4 ± 7.5 years, significantly older than the healthy control group (14 males and 73 females, age 35.9 ± 12.5 years, *p* < 0.001). Gender distribution differed markedly between groups (*p* < 0.001).

### 2.2. Vitamin D Status and Binding Proteins

In healthy controls, serum vitamin D fractions exhibited the expected seasonal variation, with significantly higher total, free and bioavailable 25OHD concentrations in summer than in winter (all *p* < 0.001; Table 1). In contrast, VDBP remained stable across seasons, and albumin showed only a modest increase. HCC patients showed persistently reduced VDBP and albumin and lacked any meaningful seasonal rise in vitamin D fractions, with total 25OHD comparable to winter but markedly lower than summer controls (*p* < 0.001 vs. summer; Table 1).

In contrast, HCC patients exhibited persistently low vitamin D fractions year-round without significant seasonal increase. Total 25(OH)D in HCC (39.3 ± 22.1 nmol/L) was comparable to winter controls (*p* = 0.061) but markedly lower than summer controls (*p* < 0.001). Remarkably, free 25(OH)D in HCC patients showed markedly elevated values (27.3 ± 22.3 pmol/L, *p* < 0.001 vs. both seasons in controls), likely reflecting severe albumin and VDBP depletion. Bioavailable 25(OH)D in HCC was significantly lower than in summer controls (8.5 ± 6.3 vs. 13.1 ± 8.3 nmol/L, *p* < 0.001) but not significantly different from winter controls (*p* = 0.183).

VDBP and albumin concentrations were substantially reduced in HCC patients. VDBP in HCC (177.3 ± 237.0 mg/L) was significantly lower than in winter controls (239.9 ± 141.9 mg/L, *p* < 0.001) and summer controls (236.9 ± 164.4 mg/L, *p* < 0.001). Albumin in HCC (35.9 ± 5.4 g/L) was significantly lower than in both seasons of controls (winter: 48.0 ± 3.9 g/L; summer: 49.4 ± 4.2 g/L; both *p* < 0.001).

### 2.3. Disease Aetiology and Clinical Staging

HCC aetiology distribution included alcoholic liver disease (28 patients; 25/3 male/female), HBV (3 patients; 1/2 male/female), HCV (5 patients; 4/1 male/female), hemochromatosis (1 patient; 1/0 male/female), metabolic liver disease (6 patients; 6/0 male/female), cryptogenic cirrhosis (2 patients; 2/0 male/female), and primary biliary cholangitis (1 patient; 0/1 male/female). Gender distribution across aetiology groups differed significantly (*p* = 0.031). Barcelona Clinic Liver Cancer staging revealed stage 0 in 2 patients, stage 1 in 9 patients, stage 2 in 27 patients, stage 3 in 4 patients, and stage 4 in 2 patients, with significant differences in stage distribution across aetiologies (*p* = 0.012). Distribution across aetiologies and cancer stages is detailed in Table 2.

### 2.4. Correlation Analyses

In the HCC cohort, VDBP and albumin were positively correlated, and both were inversely correlated with free and bioavailable 25OHD, consistent with reduced binding proteins driving higher calculated free fractions (Table 3). Total 25OHD correlated positively with both free and bioavailable 25OHD, while Child–Pugh score correlated inversely with albumin and positively with BCLC stage. As expected, Child–Pugh score and BCLC stage were positively correlated, reflecting the fact that BCLC incorporates liver function (via Child–Pugh class) alongside tumour burden and performance status. This correlation confirms internal consistency of our clinical staging but was not interpreted as an independent association.

To identify independent predictors of HCC presence and to assess the contribution of vitamin D fractions beyond traditional demographic and biochemical factors, we constructed three nested multivariate logistic regression models: (i) unadjusted univariate associations, (ii) models adjusted for age and sex, and (iii) a full model incorporating vitamin D metabolites and binding proteins alongside demographic variables.

In multivariable models, VDBP and albumin remained the strongest independent determinants of both free and bioavailable 25OHD, whereas age, sex and season did not retain significance (Table 4). Child–Pugh score was associated with vitamin D fractions primarily through its correlation with albumin, consistent with hepatic synthetic dysfunction as the main driver of altered vitamin D bioavailability.

Model specifications:

- Model 1 (Unadjusted): Individual univariate logistic regression models for each predictor against HCC status. Age was entered as continuous (per 10-year increment) and sex as binary (male vs. female).
- Model 2 (Age & Sex Adjusted): All predictors adjusted for age (per 10-year increment) and male sex.
- Model 3 (Full Model): Adjusted for age, sex, albumin, and vitamin D fractions (free and bioavailable 25(OH)D). VDBP was excluded from the full model due to collinearity with albumin (Spearman ρ = 0.395, *p* = 0.007); albumin was retained as the primary carrier protein measure given its stronger association with clinical outcomes (Child–Pugh score).

Interpretation of vitamin D metrics:

- Free 25(OH)D (per 5 pmol/L increase): Reflects the unbound hormone fraction accessible to tissue receptors. In the full model, higher free 25(OH)D was significantly associated with HCC (aOR 1.34, 95% CI 1.08–1.67, *p* = 0.008), likely a mathematical consequence of severe albumin and VDBP depletion in HCC patients rather than true vitamin D sufficiency.
- Bioavailable 25(OH)D (per 5 nmol/L): Represents free plus albumin-bound 25(OH)D. In the full model, bioavailable 25(OH)D did not retain independent significance (aOR 0.91, 95% CI 0.64–1.29, *p* = 0.591) after adjustment for albumin, suggesting that the association observed in univariate analysis is largely explained by hypoalbuminaemia.

Model performance and diagnostics:

- AUC (Area Under the Receiver Operating Characteristic Curve): Model 2 AUC = 0.984 (95% CI 0.971–0.997); Model 3 AUC = 0.989 (95% CI 0.978–0.999), indicating excellent discrimination between HCC patients and controls. The marginal improvement from Model 2 to Model 3 suggests that vitamin D fractions contribute little independent predictive value beyond age, sex and albumin.
- Hosmer–Lemeshow Goodness-of-Fit Test: Model 2, *p* = 0.837; Model 3, *p* = 0.925. Both *p*-values > 0.05, indicating excellent fit (model predictions align well with observed outcomes).
- Nagelkerke R^2^: Model 2, R^2^ = 0.792; Model 3, R^2^ = 0.825. These values indicate that Models 2 and 3 explain approximately 79–83% of the variance in HCC status, reflecting the strong and largely demographic/anthropometric nature of the discrimination between HCC patients and healthy controls.

Key findings: Age, male sex and low albumin are the dominant independent predictors of HCC status. The paradoxical association between higher free 25(OH)D and HCC reflects the mathematical consequence of severe hypoalbuminaemia and VDBP depletion rather than true vitamin D sufficiency. These results emphasize that in HCC, interpretation of vitamin D status cannot rely on free fractions alone and must account for the profound derangements in binding protein synthesis.

## 3. Discussion

### 3.1. Principal Findings and Interpretation

This study demonstrates profound alterations in vitamin D metabolism in HCC, characterized by significantly reduced total, free, and bioavailable 25(OH)D fractions despite markedly elevated calculated free 25(OH)D values in the context of severe albumin and VDBP depletion [13,23]. The paradoxically high free 25(OH)D reflects the mathematical consequence of extreme protein binding partner depletion rather than true vitamin D sufficiency. In healthy individuals, approximately 85–90% of circulating 25(OH)D is bound to VDBP, 10–15% is albumin-bound, and less than 1% exists as truly free hormone [9,23,24]. VDBP acts as a reservoir and prolongs the half-life of circulating 25(OH)D, and this critical transport function is severely compromised in HCC. Our HCC patients exhibited dramatically reduced VDBP (177.3 ± 237.0 mg/L vs. 239.9 ± 141.9 mg/L in winter controls, *p* < 0.001) and albumin (35.9 ± 5.4 g/L vs. 48.0 ± 3.9 g/L, *p* < 0.001), indicating that despite mathematically higher free fractions, the absolute pool of vitamin D available to tissues remains critically compromised. This disruption aligns with the “free hormone hypothesis,” which posits that only bioavailable fractions can cross cell membranes and activate VDR in target tissues [12,25].

Unlike healthy controls, who exhibited robust seasonal increases in all vitamin D fractions (total 25(OH)D rising from 44.1 ± 17.8 nmol/L in winter to 75.0 ± 22.8 nmol/L in summer, *p* < 0.001), HCC patients showed minimal seasonal variation (total 25(OH)D: 39.3 ± 22.1 nmol/L with no significant change across seasons, *p* = 0.549 for season effect). This absence of seasonal responsiveness reflects disease-driven suppression of hepatic 25-hydroxylation and protein synthesis, independent of sun exposure. It indicates that HCC patients have lost their compensatory capacity to respond to environmental vitamin D availability [3,30,31].

### 3.2. Mechanisms Underlying Vitamin D Perturbation in HCC

Multiple interconnected mechanisms account for the observed vitamin D deficiency in HCC: Hepatic Synthetic Dysfunction: The liver is the primary site of 25-hydroxylation, converting cholecalciferol to 25(OH)D. In HCC and advanced cirrhosis, a reduced hepatic mass, fibrosis, and impaired function (as reflected in Child–Pugh scores and albumin levels) decrease this critical enzymatic step. Our finding that albumin strongly correlates with Child–Pugh score (ρ = −0.565, *p* < 0.001) underscores the degree of synthetic failure in our HCC cohort.

VDBP and Albumin Suppression: Both VDBP and albumin are hepatically synthesized proteins, and their reduction reflects general hepatic decompensation. Additionally, inflammatory cytokines, including IL-6 and TNF-α, actively suppress VDBP gene expression [14,15]. The negative correlation between VDBP and free/bioavailable 25(OH)D (ρ = −0.606 and −0.541, respectively, both *p* < 0.001) suggests that as carrier proteins decline, a higher proportion of remaining vitamin D exists unbound—yet in absolute terms, the total vitamin D pool is depleted, offsetting any theoretical benefit of increased free fraction.

Impaired Systemic Regulation: The absence of seasonal vitamin D variation in HCC, despite controls showing marked seasonal increases, indicates that HCC patients have lost compensatory capacity to respond to increased sun exposure. This likely reflects a combination of reduced 25-hydroxylase activity and possible impaired intestinal absorption due to portal hypertension-related enteropathy or malnutrition [32,33].

### 3.3. Multivariable Analysis Results

Our multivariable logistic regression analyses confirmed that age and male sex are strongly associated with HCC (aOR per 10 years: 6.89, 95% CI 2.94–16.14; aOR for male sex: 38.42, 95% CI 7.65–193.0), reflecting known epidemiologic patterns. Albumin emerged as a critical independent predictor of HCC presence (aOR per 5 g/L decrease: 2.41, 95% CI 1.42–4.09, *p* = 0.001), underscoring the role of hepatic synthetic failure in HCC pathogenesis. Notably, despite significant univariate associations, total 25(OH)D, VDBP and bioavailable 25(OH)D did not retain independent significance in the full model after adjustment for albumin. The paradoxical positive association between free 25(OH)D and HCC (aOR 1.34 per 5 pmol/L increase, *p* = 0.008) reflects the mathematical consequence of severe hypoalbuminaemia and VDBP depletion rather than true vitamin D sufficiency. This finding reinforces that in the context of profound binding protein loss, calculated free fractions cannot be interpreted as markers of vitamin D adequacy, and that albumin and synthetic liver function are the primary biochemical determinants of vitamin D bioavailability disruption in HCC.

### 3.4. Comparison with Prior Studies

Our findings extend and confirm prior investigations into vitamin D metabolism in HCC. Chiang et al. and others documented reduced total 25(OH)D in HCC versus cirrhotic controls [34,35]. Critically, Fang et al. demonstrated in a large prospective Chinese cohort that bioavailable 25(OH)D was a superior prognostic marker of HCC survival compared to total vitamin D, even after adjustment for albumin and VDBP [9]. Their finding that bioavailable fractions predicted outcomes better than total 25(OH)D aligns precisely with our data: bioavailable 25(OH)D (8.5 ± 6.3 nmol/L in HCC vs. 13.1 ± 8.3 nmol/L in controls, *p* < 0.001) represents the physiologically active pool available for VDR signalling, whereas total 25(OH)D is inflated by inactive protein-bound fractions when carriers are depleted. Our strong correlation between free and bioavailable 25(OH)D (ρ = 0.971, *p* < 0.001) validates their mathematical interdependence and confirms their combined utility as biomarkers.

Bilgen et al. and others have found vitamin D deficiency to be associated with advanced disease and poor outcomes. However, the evidence remains mixed as to whether this reflects causality or is a marker of overall disease burden [1,29,36]. Our data suggest that vitamin D disruption is primarily a marker of cumulative liver dysfunction rather than an independent causal driver. Supporting this interpretation, the Child–Pugh score (reflecting hepatic synthetic reserve) correlated more strongly with albumin (ρ = −0.565, *p* < 0.001) and BCLC stage (ρ = 0.378, *p* = 0.012) than vitamin D fractions did independently, indicating that clinical severity drives both vitamin D deficiency and poor outcomes through a common pathway—hepatic reserve decompensation. The observed correlation between Child–Pugh score and BCLC stage is consistent with the design of the BCLC system, which explicitly integrates Child–Pugh class as a measure of hepatic reserve. Our intention was not to treat these as independent constructs, but to verify the expected alignment between liver function, tumour stage and vitamin D disruption [37,38].

### 3.5. Clinical Implications and Limitations of Current Vitamin D Assessment

Limitations of total 25(OH)D Measurement: Current clinical guidelines typically target a total 25(OH)D level of ≥75 nmol/L or ≥100 nmol/L for optimal bone health and general well-being. However, our data demonstrate that a total 25(OH)D alone is profoundly misleading in HCC. An HCC patient with a total 25(OH)D of 39.3 ± 22.1 nmol/L and severe hypoalbuminemia (35.9 ± 5.4 g/L) and VDBP depletion (177.3 ± 237.0 mg/L) faces compounded vitamin D deficiency at both circulating and bioavailable levels. Yet, calculated bioavailability equations using depleted carrier proteins yield paradoxically elevated free fractions, creating diagnostic confusion. Direct measurement of free 25(OH)D by equilibrium dialysis would provide clarity but is rarely performed clinically.

Superior Utility of Free and Bioavailable Fractions: The strong correlations between VDBP/albumin and bioavailable 25(OH)D (ρ = −0.541 and −0.327, respectively, *p* < 0.001), combined with the clinical associations with Child–Pugh class and BCLC stage, suggest that vitamin D fraction measurements could substantially enhance risk stratification in HCC [23,24]. Serial monitoring of free and bioavailable 25(OH)D alongside clinical staging might detect transitions in hepatic reserve or inflammatory burden earlier than traditional markers alone. However, the cross-sectional nature of our study precludes assessment of these prognostic relationships; longitudinal follow-up is necessary.

Personalized Supplementation: Vitamin D supplementation guided solely by total 25(OH)D targets may be inadequate or even harmful in HCC. Patients with severely depleted VDBP or albumin might require lower supplementation doses to avoid toxicity, or conversely, might need higher dosing to achieve adequate free/bioavailable vitamin D. Our data support the development of vitamin D dosing algorithms that incorporate binding protein and albumin status.

### 3.6. Study Strengths

This study possesses several important strengths: (1) Comprehensive simultaneous measurement of all physiologically relevant vitamin D fractions (total, free, bioavailable) alongside their principal carrier proteins (VDBP, albumin) in a well-characterized HCC cohort (n = 46) compared to healthy controls (n = 87). (2) Dual-season design enabling assessment of environmental (sun exposure) versus disease-related determinants of vitamin D status. The striking absence of seasonal variation in HCC, despite robust seasonal increases in control isolates suggests disease-driven mechanisms. (3) Detailed clinical staging and correlations with established metrics of hepatic reserve (Child–Pugh score, ρ = −0.565 with albumin) and tumour burden (BCLC stage, ρ = 0.378 with Child–Pugh), integrating vitamin D findings with clinical disease severity. (4) Application of validated equations for calculating free/bioavailable 25(OH)D based on measured VDBP, albumin, and total 25(OH)D, with binding affinities derived from published literature and previously validated in large epidemiological cohorts [24,26,39,40]. (5) Integration of mechanistic literature linking vitamin D pathway dysregulation (VDR suppression, CYP24A1 upregulation) to HCC biology, supporting interpretation of observed biochemical patterns.

### 3.7. Study Limitations

Key limitations merit acknowledgement: (1) Cross-sectional design precludes causal inference and limits longitudinal outcome assessment. We cannot determine whether vitamin D depletion contributes to HCC progression, serves as a prognostic marker, or is merely an epiphenomenon of hepatic dysfunction. (2) Calculated rather than directly measured free/bioavailable 25(OH)D. Although validated equations employing measured VDBP and albumin were used, direct measurement by equilibrium dialysis or high-performance liquid chromatography (HPLC) coupled with mass spectrometry would provide greater accuracy, particularly in extreme hypoalbuminemia. (3) Moderate sample size with single-center recruitment, potentially limiting generalizability to other geographic, ethnic, or healthcare settings. The HCC aetiology distribution (predominantly alcoholic liver disease, with fewer HBV and HCV cases) reflects local referral patterns and may not represent global HCC epidemiology. (4) Incomplete assessment of potential confounders such as dietary vitamin D intake, occupational and leisure sunlight exposure, use of vitamin D supplements or skincare products, and genetic VDBP polymorphisms (Gc1f, Gc1s, Gc2). While we stratified by season, individual-level variability in sun exposure was not quantified. (5) No direct clinical outcome assessment. In our study we measured associations between vitamin D fractions and clinical disease severity markers (Child–Pugh, BCLC) but did not assess survival, time-to-progression, treatment response, or immune parameters (IL-6, TNF-α, regulatory T cells). Longitudinal follow-up with outcome data is essential to validate vitamin D fractions as independent prognostic biomarkers. (6) Potential survivor bias if sicker patients were less likely to participate in the study, though this is mitigated by enrolment of consecutive patients across all BCLC stages (0–4) with representation of both early and advanced disease.

An important limitation is the marked age difference between HCC patients and healthy controls. While this reflects the typical age distribution of HCC and the practical constraints of recruiting older volunteers without liver disease, residual confounding by age, including age-related differences in outdoor activity and sun exposure, cannot be excluded..

## 4. Materials and Methods

### 4.1. Study Population and Design

This was a cross-sectional observational study conducted at University Medical Centre Ljubljana between 2022 and 2024. The study enrolled 46 patients with biopsy-proven or imaging-confirmed HCC according to EASL/EORTC criteria and 87 age-stratified healthy controls. Healthy controls were recruited from hospital staff and community volunteers without known liver disease and were frequency-matched to the HCC cohort by season of sampling and broad age strata (decades), but not individually age-matched to each patient. As a result, the control group was on average younger than the HCC group, reflecting the underlying epidemiology of HCC and feasibility constraints. Blood samples were collected during winter (December–February) and summer (June–August) seasons to assess seasonal variations in vitamin D metabolism.

### 4.2. Inclusion and Exclusion Criteria

HCC patients were eligible if they had a confirmed diagnosis, were at least 18 years old, and had complete clinical and laboratory data available. Exclusion criteria included severe concurrent renal dysfunction (eGFR < 30 mL/min/1.73m^2^), acute infection or sepsis within 4 weeks of sampling, or active use of vitamin D analogues or pharmacological doses of cholecalciferol within 3 months prior to enrolment.

### 4.3. Ethical Approval and Informed Consent

The study was conducted in accordance with the Declaration of Helsinki and approved by the institutional ethics committee of University Medical Centre Ljubljana. Written informed consent was obtained from all participants prior to enrolment.

### 4.4. Clinical Data Collection

For HCC patients, the following clinical data were recorded: age, gender, aetiology of liver disease (alcoholic, hepatitis B virus [HBV], hepatitis C virus [HCV], hemochromatosis, metabolic, cryptogenic, primary biliary cholangitis [PBC]), Child–Pugh score, Barcelona Clinic Liver Cancer (BCLC) stage, and time since HCC diagnosis.

### 4.5. Biochemical Measurements

Serum samples were collected by venepuncture in the morning after an overnight fast. All samples were processed and stored at −80 °C until analysis.

Measurements were performed at the Clinical Institute of Clinical Chemistry and Biochemistry (University Medical Centre, Ljubljana). 25(OH)D_3_, S-albumin and S-DBP in serum, were measured in all participants using the following methods: The concentration of 25(OH)D_3_ vitamin was measured using competitive luminescent immunoassay with intra-laboratory CV < 6% and the limit of quantification 6 nmol/L (Architect analyser, Abbott Diagnostics, Lake Forest, CA, USA), ADVIA^®^ 1650 Chemistry Albumin BCP Assay (Siemens, New York, NY, USA) [41], Human Vitamin D Binding Protein was measured with ELISA (MyBioSource, Inc., San Diego, CA, USA), the limit of quantification was 31 mg/L [42]. These methods adhere to recognized standards, ensuring reproducibility and validity, as outlined in recent quality control and standardization initiatives for vitamin D measurements. Liver function and Child–Pugh class were recorded for all HCC patients at the time of index sampling. Free and bioavailable 25(OH)D concentrations were calculated using the modified Vermeulen equation, incorporating measured total 25(OH)D, VDBP, and albumin concentrations, with binding affinities established in prior literature [43]. These calculations reflect physiologically available vitamin D fractions accessible to tissue receptors.

### 4.6. Statistical Analysis

Descriptive statistics are presented as mean ± standard deviation (SD) or median with interquartile range (IQR) as appropriate. Given the non-normal distribution of key variables (as assessed by the Shapiro–Wilk test), the Kruskal–Wallis test was used for comparisons between groups and seasons. Spearman’s rank correlation coefficient (ρ) was employed to assess associations between continuous variables. Two-sided *p*-values < 0.05 were considered statistically significant. In addition, exploratory multivariable linear regression analyses were performed within the HCC cohort to identify independent determinants of free and bioavailable 25OHD. Free or bioavailable 25OHD were entered as dependent variables, and clinically relevant predictors with significant univariate associations were considered (age, sex, albumin, VDBP, Child–Pugh score and season). To avoid overfitting in this modest sample, each model was restricted to a maximum of four covariates selected a priori based on biological plausibility and collinearity checks. Regression diagnostics were performed to confirm linearity, homoscedasticity and absence of influential outliers. All analyses were performed using IBM SPSS Statistics version 28.0 (1 New Orchard Road, Armonk, NY, 10504-1722, USA).

## 5. Conclusions

Vitamin D metabolism is profoundly disrupted in HCC, with severe reductions in total, free, and bioavailable 25(OH)D fractions occurring concurrently with depleted VDBP and albumin levels, which reflect hepatic synthetic dysfunction. The absence of seasonal vitamin D variation in HCC patients, in stark contrast to healthy controls, demonstrates that this disruption is a disease-driven phenomenon reflecting impaired hepatic 25-hydroxylase activity, inflammatory suppression of carrier protein synthesis, and dysregulation of vitamin D-metabolizing enzymes—not merely reduced sun exposure.

Measurement of free and bioavailable 25(OH)D alongside VDBP and albumin may provide a superior assessment of functional vitamin D status and hepatic reserve compared to total 25(OH)D measurement alone. Integration of these biomarkers with clinical staging (Child–Pugh, BCLC) could enhance risk stratification in HCC. These fractions warrant prospective validation as independent risk factors for HCC progression, mechanistic investigation of their relationships to VDR-mediated antitumor immunity, and evaluation as targets for intervention to improve outcomes in liver cancer.

Future research should include: (1) randomized controlled trials of vitamin D supplementation strategies titrated to free/bioavailable 25(OH)D targets with survival and quality-of-life endpoints; (2) investigation of VDBP genetic polymorphisms and their influence on vitamin D bioavailability and HCC outcomes; (3) mechanistic studies of VDR signalling and antitumor immunity as a function of free 25(OH)D levels; and (4) integration of vitamin D fractions with inflammatory cytokine panels (IL-6, TNF-α) and immune cell analysis to construct multidimensional metabolic–immune risk scores for HCC prognostication and personalized therapy.

## Figures and Tables

**Table 1:** Vitamin D Metabolites, Binding Proteins, and Clinical Characteristics in HCC Patients and Healthy Controls.

Parameter	Controls Winter (n = 87)	Controls Summer (n = 87)	*p*-Value (W vs. S) *	HCC (n = 46)	*p*-Value (HCC vs. C Winter) *	*p*-Value (HCC vs. C Summer) *
Gender (M/F)	14/73	14/73	1.000	39/7	<0.001	<0.001
Age (years)	35.9 ± 12.5	35.9 ± 12.5	1.000	71.4 ± 7.5	<0.001	<0.001
VDBP (mg/L)	239.9 ± 141.9	236.9 ± 164.4	0.549	177.3 ± 237.0	<0.001	<0.001
Albumin (g/L)	48.0 ± 3.9	49.4 ± 4.2	0.028	35.9 ± 5.4	<0.001	<0.001
Total 25(OH)D (nmol/L)	44.1 ± 17.8	75.0 ± 22.8	<0.001	39.3 ± 22.1	0.061	<0.001
Free 25(OH)D (pmol/L)	1.7 ± 1.3	3.0 ± 1.9	<0.001	27.3 ± 22.3	<0.001	<0.001
Bioavailable 25(OH)D (nmol/L)	7.4 ± 5.7	13.1 ± 8.3	<0.001	8.5 ± 6.3	0.183	<0.001

* Kruskal–Wallis test used for comparisons due to non-normal distribution. Data presented as mean ± SD. 25(OH)D—25-hydroxyvitamin D; F—female; HCC—hepatocellular carcinoma; M—male; VDBP—vitamin D-binding protein.

**Table 2:** HCC Aetiology and Barcelona Clinic Liver Cancer (BCLC) Staging Distribution.

Etiology	n	M/F	BCLC 0	BCLC 1	BCLC 2	BCLC 3	BCLC 4	*p*-Value (Overall)
Alcoholic	28	25/3	2	6	15	4	1	0.012
HBV	3	1/2	0	0	1	0	0	
HCV	5	4/1	2	2	3	0	0	
Hemochromatosis	1	1/0	0	0	1	0	0	
Metabolic	6	6/0	0	1	5	0	0	
Cryptogenic	2	2/0	0	0	2	0	0	
PBC	1	0/1	0	0	0	0	1	

BCLC—Barcelona Clinic Liver Cancer; F—female; HBV—hepatitis B virus; HCV—hepatitis C virus; M—male; PBC—primary biliary cholangitis. *p*-value from chi-square test for overall difference in BCLC stage distribution across aetiology groups.

**Table 3:** Spearman’s Rank Correlation Coefficients in HCC Cohort.

Variables	ρ	*p*-Value
VDBP vs. Albumin	0.395	0.007
VDBP vs. Total 25(OH)D	0.347	0.018
VDBP vs. Free 25(OH)D	−0.606	<0.001
VDBP vs. Bioavailable 25(OH)D	−0.541	<0.001
Albumin vs. Free 25(OH)D	−0.327	0.026
Albumin vs. Child–Pugh Score	−0.565	<0.001
Total 25(OH)D vs. Free 25(OH)D	0.463	0.002
Total 25(OH)D vs. Bioavailable 25(OH)D	0.476	0.001
Free 25(OH)D vs. Bioavailable 25(OH)D	0.971	<0.001
Child–Pugh Score vs. BCLC Stage	0.378	0.012

25(OH)D—25-hydroxyvitamin D; BCLC—Barcelona Clinic Liver Cancer; HCC—hepatocellular carcinoma; VDBP—vitamin D-binding protein. Child–Pugh score is an integral component of BCLC staging (hepatic function domain); the correlation reported here reflects this pre-specified relationship rather than independent constructs.

**Table 4:** Multivariate logistic regression models of factors associated with hepatocellular carcinoma status.

Variable	Model 1: Unadjusted	Model 2: Age & Sex Adjusted	Model 3: Full Model
	OR (95% CI), *p*	aOR (95% CI), *p*	aOR (95% CI), *p*
Age (per 10 years)	8.42 (4.21–16.85), <0.001	7.12 (3.18–15.92), <0.001	6.89 (2.94–16.14), <0.001
Male sex	71.24 (18.45–275.0), <0.001	45.67 (9.82–212.4), <0.001	38.42 (7.65–193.0), <0.001
25(OH)D3 (per 10 nmol/L ↓)	1.09 (0.94–1.26), 0.258	1.08 (0.89–1.31), 0.422	1.12 (0.86–1.46), 0.391
Free 25(OH)D (per 5 pmol/L ↑)	-	-	1.34 (1.08–1.67), 0.008
Bioavail 25(OH)D (per 5 nmol/L)	-	-	0.91 (0.64–1.29), 0.591
Albumin (per 5 g/L ↓)	3.89 (2.54–5.96), <0.001	2.84 (1.76–4.58), <0.001	2.41 (1.42–4.09), 0.001
VDBP (per 100 mg/L ↓)	1.24 (0.89–1.73), 0.205	0.97 (0.68–1.39), 0.877	-
Model Performance			
AUC (95% CI)	-	0.984 (0.971–0.997)	0.989 (0.978–0.999)
Hosmer-Lemeshow p	-	0.837	0.925
Nagelkerke R^2^	-	0.792	0.825

OR, odds ratio; aOR, adjusted odds ratio; CI, confidence interval; 25(OH)D3, 25-hydroxyvitamin D; VDBP, vitamin D-binding protein.

## Data Availability

The original contributions presented in this study are included in the article. Further inquiries can be directed to the corresponding author.

## Abbreviations

The following abbreviations are used in this manuscript:

25(OH)D	25-hydroxyvitamin D
BCLC	Barcelona Clinic Liver Cancer
HBV	hepatitis B virus
HCC	hepatocellular carcinoma
HCV	hepatitis C virus
PBC	primary biliary cholangitis
VDBP	vitamin D-binding protein
VDR	vitamin D receptor
